# Structure of Diamond Films Grown Using High-Speed Flow of a Thermally Activated CH_4_-H_2_ Gas Mixture

**DOI:** 10.3390/ma13010219

**Published:** 2020-01-04

**Authors:** Yu.V. Fedoseeva, D.V. Gorodetskiy, K.I. Baskakova, I.P. Asanov, L.G. Bulusheva, A.A. Makarova, I.B. Yudin, M.Yu. Plotnikov, A.A. Emelyanov, A.K. Rebrov, A.V. Okotrub

**Affiliations:** 1 Nikolaev Institute of Inorganic Chemistry SB RAS, 3 Acad. Lavrentiev Ave., 630090 Novosibirsk, Russia; 2 Novosibirsk State University, 2 Pirogova Str., 630090 Novosibirsk, Russia; 3 Physical Chemistry, Institute of Chemistry and Biochemistry, Free University of Berlin, 14195 Berlin, Germany; anna.makarova@helmholtz-berlin.de; 4 Kutateladze Institute of Thermophysics, SB RAS, 1 Acad. Lavrentiev Ave., 630090 Novosibirsk, Russia

**Keywords:** diamond film, high-speed gas flow, jet-deposition, surface coating, XPS, NEXAFS

## Abstract

Diamond films are advanced engineering materials for various industrial applications requiring a coating material with extremely high thermal conductivity and low electrical conductivity. An approach for the synthesis of diamond films via high-speed jet deposition of thermally activated gas has been applied. In this method, spatially separated high-speed flows of methane and hydrogen were thermally activated, and methyl and hydrogen radicals were deposited on heated molybdenum substrates. The morphology and structure of three diamond films were studied, which were synthesized at a heating power of 900, 1700, or 1800 W, methane flow rate of 10 or 30 sccm, hydrogen flow rate of 1500 or 3500 sccm, and duration of the synthesis from 1.5 to 3 h.The morphology and electronic state of the carbon on the surface and in the bulk of the obtained films were analyzed by scanning electron microscopy, Raman scattering, X-ray photoelectron, and near-edge X-ray absorption fine structure spectroscopies. The diamond micro-crystals with a thick oxidized amorphous *sp*^2^-carbon coating were grown at a heating power of 900 W and a hydrogen flow rate of 1500 sccm. The quality of the crystals was improved, and the growth rate of the diamond film was increased seven times when the heating power was 1700–1800 W and the methane and hydrogen flow rates were 30 and 3500 sccm, respectively. Defective octahedral diamond crystals of 30 μm in size with a thin *sp*^2^-carbon surface layer were synthesized on a Mo substrate heated at 1273 K for 1.5 h. When the synthesis duration was doubled, and the substrate temperature was decreased to 1073 K, the denser film with rhombic-dodecahedron diamond crystals was grown. In this case, the thinnest hydrogenated *sp*^2^-carbon coating was detected on the surface of the diamond crystals.

## 1. Introduction

The development of new technical strategies for the rapid synthesis of high-quality diamond films remains an attractive and challenging task. A combination of properties, namely good electrical insulation, high hardness, excellent thermal conductivity, low dielectric constant, and transparency in a wide wavelength range makes diamonds well suited for mechanical, thermal, optical, and electronic applications [1,2,3,4]. Thin diamond films can be used as a coating for cutting tools [5], fiber-optic low coherence sensors [6], biosensors [7], ultraviolet photodetectors [4], etc. The conductive diamond films can be applied as an electrode for electrochemistry, electrosynthesis, and electrocatalysis [8]. Polycrystalline diamond films synthesized usingthe energy-assisted chemical vapor deposition (CVD) method enable these properties to be optimized, controlled, and exploited for various purposes [3,9]. The CVD technique is based on the dissociation of carbon-containing precursor molecules on the surface of a solid substrate and the subsequent reaction between the decomposition products with the formation of a diamond film.

For activation of carbon-containing gas, thermal methods (hot-filament) or electric discharge (DC, RF, or microwave) are usually applied. Thermal activation of precursor gases by a hot filament (HF) is widely used to produce synthetic diamond structures [1,10,11,12,13]. The advantages of this method are its low cost, simplicity, scalability, and ability to use it to coat surfaces of complex relief. In HF CVD, the precursor gases, usually a mixture of CH_4_ and H_2_ is activatedby a carburized tungsten or tantalum filament that is heated by an electric current to a temperature of approximately 2500 K. The main role of the hot-filament is to generate methyl radicals, which are precursors of diamond phase growth, and atomic hydrogen. Atomic hydrogen was found to etch graphite co-deposits much faster than diamond species, thus promoting the growth of large diamond crystals [14]. However, due to the upper temperature limit of the filament material, the concentration of atomic hydrogen in HF CVD syntheses is significantly lower than that in plasma-activated CVD processes. The higher filament temperature is better for the diamond growth, but the carbon film formed on the metallic surface of the hot filament deactivates it [10]. Moreover, the diamond growth rate depends on the ability of hydrocarbon and hydrogen free radicals to be transported to the substrate before their spontaneous recombination.

In the classical HF CVD method, the diamond growth rate is limited by the number of collisions of molecules with the hot filament and diffusion of the dissociated species to a substrate. Moreover, partial cooling of active particles occurs near the cold surface of the substrate. The HF CVD method provides relatively low growth rates (1–15 μm/h) as compared with plasma-chemical methods (<200 μm/h) [10,15,16,17,18]. In recent years, gas-jet modification of the HF CVD method has been actively developed. The advantages and problems of the gas-jet process for the growth of diamond using heated tungsten channels are described in [13,19,20,21,22,23,24]. A distinctive feature of this approach is the use of heterogeneous dissociation processes during multiple collisions of molecules with a hot surface [19,25]. High-speed supply of precursor gases through a hot filament allows increasing the concentration of dissociated species because of multiple collisions of gas molecules with the hot surface and some freezing of chemical reactions inside the jet. The jet increases the feed rate of high-energy particles, especially heavy carbon-containing ones, to the substrate, which can be additionally heated to increase the concentration of carbon and improve atomic connectivity in the diamond film. The gas-jet method can significantly increase the energy of the interaction of carbon-containing species with the heated substrate due to their acceleration in the jet. The gas-jet approach allows one to provide deposition conditions different from the classical HF CVD method, where the delivery of active components to the substrate occurs due to their diffusion, allowing an increase in the growth rate of the diamond phase. Here we use the newly developed design of an experimental gas-jet reactor in which high-speed jets of methane and hydrogen were fed separately through the internal and external channels of a hot spiral tungsten filament [22]. Such separate activation of gases allows producing a high concentration of atomic hydrogen and hydrocarbons at extremely high temperatures (about 2700 K).

Numerous studies about HF CVD syntheses of diamond films with micro- or nano-crystalline diamond particles, a columnar structure, or individual particles can be found in the literature [26,27,28]. The nucleation density, size, and crystallographic morphology of particles usually depend on the synthesis conditions. In the case of the gas-jet variant of the HF CVD technique, there is no experimental experience in the deposition of diamond films with different sizes and shapes. Thus, the question of adjustment of the scheme of this synthesis to produce the film with a specific morphology requires further research. Our previous study revealed that the gas-jet deposition using jets of CH_4_-H_2_ mixtures results in formation of diamond particles with an average size of 30 μm and a rhombic dodecahedral shape [24]. The small particles with sizes between 10 and 100 nm were found to be located on the surface of the microcrystals and in the crevices between the well-formed diamond planes. The diamond films consisting of both micro- and nanocrystals exhibit better adhesive properties due to the interfaces formed between the crystals [29]. Since the high speed of the diamond growth is one of the advantages of the gas-jet technique, the predominantly produced microcrystalline diamond films can be used as a wear-resistance coating for cutting tools or for heat dissipation in electrical appliances, as well as for heat resistance substrates. For improving the adhesion of the diamond film, we used the Mo substrate since molybdenum has a coefficient of linear expansion (5 × 10^−6^ K^−1^) similar to that for diamond (~10^−6^ K^−1^) [30,31], and a high density of nucleation centers (10^8^ cm^−2^) [32,33].

The growth rate, morphology, atomic, and crystalline structure of diamond films produced at different power of thermal activation, methane and hydrogen flow rates, duration of synthesis, and substrate temperature have been studied using a set of modern non-destructive characterization tools. The surface composition and chemical bonding have been investigated in details by X-ray photoelectron spectroscopy (XPS) and near-edge X-ray absorption fine structure (NEXAFS) spectroscopy. The surface state of the diamond crystals is one of the determining factors that affect some functional properties, for example, optical transparency and electrical conductivity.

## 2. Materials and Methods

### 2.1. Synthesis

**Figure 1:**
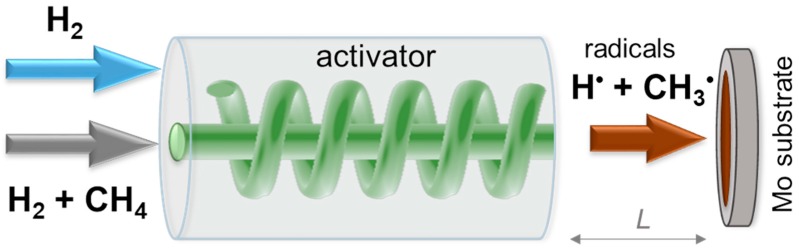
Scheme of the synthesis process of diamond films using the thermally activated gas-jet method.

The synthesis of diamond films was performed by the gas-jet synthesis method in an experimental setup described in detail elsewhere [22]. The principle of operation of the experimental setup is shown in Figure 1. A high-speed jet of methane mixed with hydrogen was injected into the inner channel of a cylindrical reactor for thermal activation of gases. The rate of methane flow (R_m_) was varied from 10 to 30 sccm, and the hydrogen flow rate was 1500 sccm. The hydrogen jet with a flow rate at 1500 or 3500 sccm was supplied simultaneously to an annular channel located outside of the activator. The spiral-shaped reactor made of tungsten resistively heated the jet of the gases at a power (P) of 900, 1700 or 1800 W. The temperature of the activator was estimated to reach 2400 K for 900 W, 2600 K for 1700 W, and 2700 K for 1800 W. The separate injection of methane and hydrogen was used to prevent carbidization of the tungsten activator;that allowed us to increase the heating power and consequently the concentration of H atoms. Thermal activation of methane and hydrogen jets yielded a high-speed flow of H atoms and CH_x_ radicals, which were required for successful diamond growth.

The flow of the activated gas mixture was deposited on a transverse Mo substrate at temperature T_s_. The small distance between the reactor and the substrate (L=1 cm) and the rapid transfer of radicals to the metal surface can significantly increase the diamond growth rate. Pure molybdenum foil with a diameter of 20 mm and a thickness of 250 μm, or that covered with nanodiamond seeds using an ultrasonically treated suspension, were used as substrates. The pressure in the reactor was 20 Torr during experiments. Three samples were synthesized at different parameters, such as heating power of the activator, external hydrogen flow ratio, methane flow ratio, temperatures of the heater and substrates, synthesis duration, and nanodiamond seeds on the substrates (Table 1).

### 2.2. Characterization

Morphology of the samples was investigated by scanning electron microscopy (SEM) on aS-3400N (Hitachi Ltd., Berkshire, UK) microscope and on a JSM 6700F (JEOL Ltd., Tokyo, Japan) microscope. The images were obtained at 20 kV accelerating voltage in secondary electrons mode. Raman spectra were measured with a LabRAM HR Evolution (Horiba, Kyoto, Japan) spectrometer using the Ar^+^ laser with a wavelength of 514 nm. XPS spectra were recorded on a Phoibos 150 (SPECS GmbH, Berlin, Germany) spectrometer using a monochromatized Al *K_α_* radiation (1486.7 eV). The pass energy of the electron energy analyzer was set at 20 eV. The base pressure for the measurements was ~10^−9^ mbar. The C 1s-spectra were fittedviaGaussian–Lorentzian functions with a full-width at half-maximum (FWHM) of 1.5–1.7eV. NEXAFS experiments were performed at Russian–German beamline at the Berliner ElektronenspeicherringfürSynchrotronstrahlung (BESSY II), the Helmholtz-Zentrum Berlin (Germany)using monochromatized synchrotron radiation in the soft X-ray region. NEXAFS spectra near the C K-edge were acquired in the total electron yield (TEY) and Auger electron yield (AEY) modes.

## 3. Results

**Figure 2:**
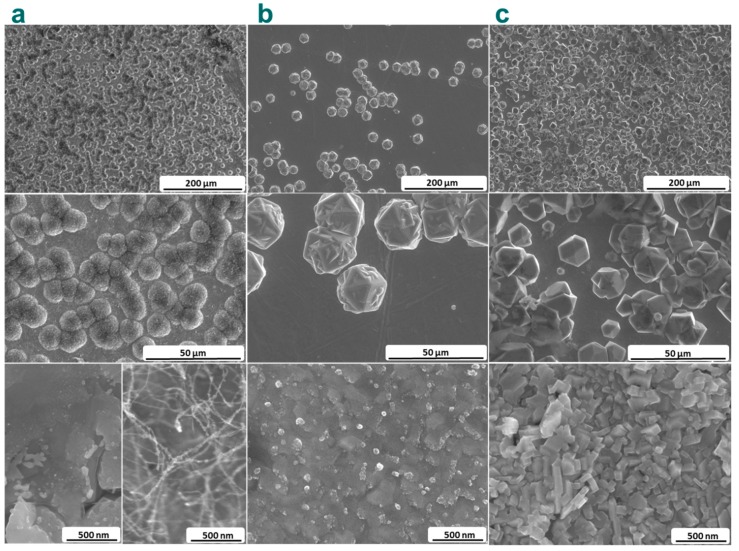
SEM images of the surface of diamond films: Sample 1 (**a**), Sample 2 (**b**) and Sample3(**c**), grown at different parameters of gas-jet synthesis. Bottom images were taken in the spaces between microparticles.

Figure 2 shows SEM images of the surface of the diamond films produced at different synthesis parameters. In all cases, micro-sized particles of different shapes unevenly covered the surfaces of the Mo substrates. Sample 1 consisted of spherically shaped microparticles with surface carbon flakes (Figure 2a). The sizes of these agglomerates are less than 10μm. Since nanodiamond seeds were used during the synthesis, it can be suggested that products of the gas-jet synthesis covered these grains during the growth process. Note that the particles of the shapes characteristic for diamond crystals were not observed in Sample 1. That means that conditions used for the synthesis of Sample 1 did not promote nucleation and growth of large diamond crystals. However, the crystals have been grown successfullyin Samples 2 and 3 (Figure 2b,c). The biggest octahedral diamond crystals of *ca*.30 μm in size and without a visible amorphous phase were found in Sample 2 (Figure 2b). Moreover, grown together diamond crystals and secondary nucleation on the surface side of the diamond octahedrons were observed in this sample. The high-density film consisted of almost perfect diamond crystals was identified in Sample 3 (Figure 2c). Here the diamond crystals had a rhombic-dodecahedron shape and the largest variation in size from 5 to 30 μm. The presence of small crystals indicates that diamond nucleation can occur continuously. Most crystals stacked together covering almost all area of the substrate surface. Diamond growth rate was calculated as the ratio between the diameter of the largest particle and the synthesis duration. The growth rate for Sample 1 is 3 μm/h and it reaches 20 and 10 μm/h for Samples 2 and 3, respectively.According to SEM images, microcrystals of diamonds did not fully cover the substrate surface. Despite that fact, the concentration of Mo on the surface of the samples, which was estimated from the intensity of Mo 3d line in the overall XPS, is less than 4 at%. This indicates a continuous coating of the substrate by carbon. Actually, SEM images taken with higher magnification revealed the coating consisting of 10–500 nm nanodiamonds between the microparticles (Figure 2, bottom images). The formation of carbon fibers was observed in Sample 1, while mainly cubic diamond particles completely covered the substrate.

**Figure 3:**
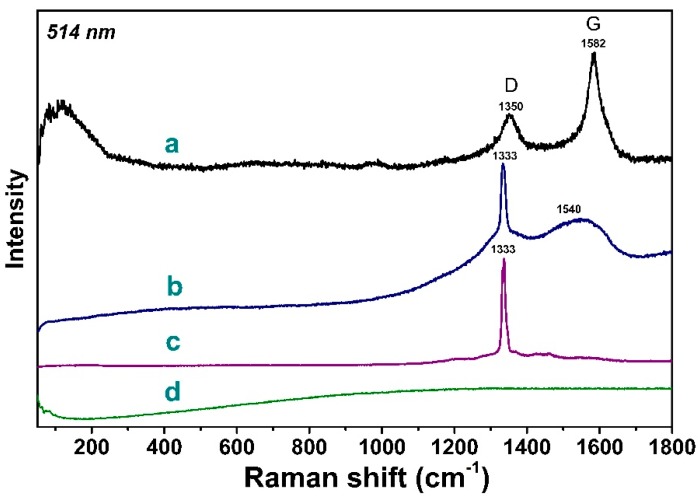
Raman spectra of Sample 1 (**a**), Sample 2 (**b**), and Sample 3 (**c**) grown at different parameters of gas-jet synthesisin comparison with the spectrum of initial Mo substrate (**d**).

Raman spectra of samples measured at 514 nm wavelength are compared in Figure 3. The spectrum of Sample 1 showed the G-mode peak at 1582 cm^−1^, which originates from stretching vibrations of the bonds between *sp*^2^-hybridized carbon atoms, and a disorder-induced D-mode peak at 1350 cm^−1^ (Figure 3a). These two peaks are typical for materials containing *sp^2^*-carbon, graphitic-like or amorphous [34,35,36]. According to published data, similar Raman features were observed in the diamond thin film grown on silicon usingthe HF CVD method [37] and diamond-like films produced by HF CVD and plasma-enhanced (PE) CVD with the addition of high argon concentration [38,39]. In the case of polycrystalline diamond films, graphitic-like carbon can be located on the surface of diamond particles. The high intensity of the G- and D-peaks in the spectrum of Sample 1 indicates that the flakes that cover the spherical particles are composed of *sp*^2^-hybridized carbon. A narrow intense peak at 1333 cm^−1^ arisen from the diamond crystal structure [40] dominated in Raman spectra of Samples 2 and 3 (Figure 3b,c). The spectrum of Sample 2 additionally showed weak wide peaks at around 1540 cm^−1^, a wide band between 1250 and 1400 cm^−1^, and a sloping background from photoluminescence. The broad band at 1540 cm^−1^ is usually attributed to diamond-like carbon [41], while the wide band at 1250–1400 cm^−1^ could be assigned to amorphous *sp^3^*-bonded carbon [42]. The full width at half maximum (FWHM) of the 1333 cm^−1^ diamond peak was estimated to be 15 cm^−1^ for Sample 2 and 11 cm^−1^ for Sample 3. The smaller peak width for Sample 3 is more probably due to the better crystal structure of the diamond particles. This value is similar to the FWHM of 4.7–11.6 cm^−1^ observed for microwave PE CVD diamond films [43]. The absence of any peaks at 1500–1600 cm^−1^ indicates that Sample 3 is high-quality diamond films with minimal quantities of the non-diamond components.

Raman spectroscopy provides information on molecular vibrations and local crystal structure of the samples, while XPS and NEXAFSare the best tools for probing the composition and local atomic environment of elements and chemical bonding. To study the chemical states of carbon atoms located in the bulk of diamond particles, we measured NEXAFS C K-edge spectra in TEY mode providing a probing depth of about 10 nm [44,45]. The NEXAFS spectroscopy in AEY mode has a much lower probing depth because the inelastic mean free path of low-energy Auger electrons is less than *ca*.1 nm. Thus, in combination with XPS it was used to establish the presence of graphitic carbon and other chemical functionalities on the surface of diamond crystals [46,47].

**Figure 4:**
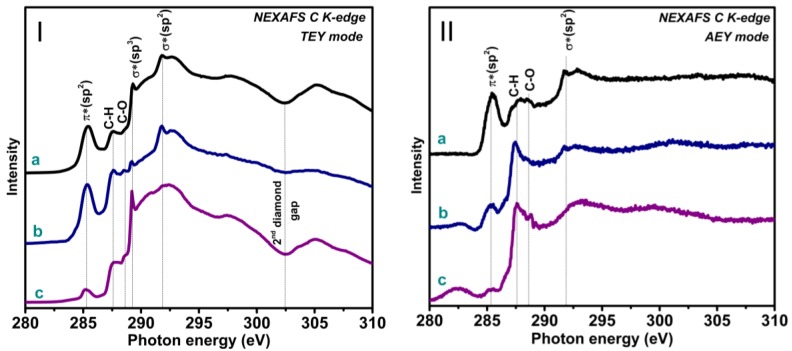
NEXAFS C K-edge spectra of Sample 1 (**a**), Sample 2 (**b**), and Sample3(**c**) grown at different parameters of gas-jet synthesis. The spectra were measured in TEY (**I**) and AEY (**II**) modes.

The NEXAFS C K-spectra of samples measured in TEY mode (Figure 4I) exhibited a peak at 289.4 eV arising from 1*s*→σ* electron transitions within the *sp*^3^–hybridized diamond carbon and a gap at 302.5 eV corresponding to the second absolute gap in the diamond band structure [48]. The presence of these diamond features in NEXAFS spectra confirms the formation of diamond crystals in all samples. The absence of the diamond peak in the Raman spectrum of Sample 1 indicates a small size of diamond crystals and their poorly ordered structure overall. A high intensity and sharpness of the diamond resonance observed in the spectrum of Sample 3 (Figure 4Ic) confirm the formation of big and high-quality crystals. The features observed in the NEXAFS C K-spectra at 285.4 and 291.7 eV correspond to electron transitions from 1s levels to π* and σ* states within the *sp*^2^-hybridized carbon atoms [49]. Two features at 286 and 287 eV arise from hydrogenated and oxygenated carbon atoms [50]. Contributions by non-diamond carbon were significant in the spectra of Samples 1 and 2 (Figure 4Ia,b) and minimal in the spectrum of Sample 3 (Figure 4Ic). NEXAFS spectra measured in AEY mode (Figure 4II) did not reveal spectral features from the diamond phase on the surface of all studied samples. However, intense features arisen from *sp*^2^-carbon and hydrogenated and oxygenated carbon were observed. That confirms the location of these species on the surface of the diamond crystals. It is notable that such surface carbon hasa different composition in the samples produced under different conditions. Graphitic-like carbon is responsible for the intense peak at 285.4 eV in the spectrum of Sample 1 (Figure 4IIa), but its concentration decreases in other samples and most substantially in Sample 3 (Figure 4IIc). The peaks corresponding to surface C–H and C–O bonds had comparable intensities in the spectrum of Sample 1; however, the former peak dominated in the spectra of Samples 2 and 3.

**Figure 5:**
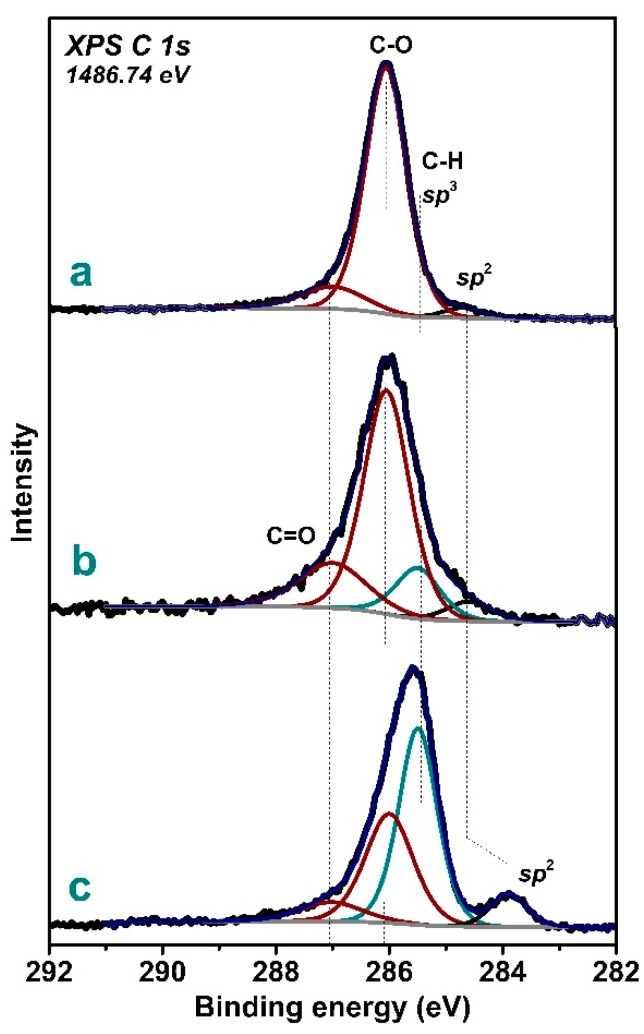
XPS C 1s spectra of Sample 1 (**a**), Sample 2 (**b**), and Sample 3 (**c**) grown at different parameters of gas-jet synthesis.

The XPS C 1*s* spectra measured at 1486.74 eV examined the samples to a depth of less than 10 nm [51]. Four components fitted the spectra (Figure 5). The peak at 284.0–284.5 eV corresponds to *sp*^2^-hybridized graphitic-like and/or amorphous carbon growing together with the diamond phase in most experimental cases [38,52]. The component at 285.3 eV is attributed to carbon atoms in the *sp*^3^-hybridized state, realizing in both the diamond phase and hydrogenated diamond surface [24,53]. The oxygen-containing surface ether and hydroxide groups give a C–O peak at 286.0 eV, while the C=O peak at 287.5 eV arises from carbonyl groups [54,55]. Analysis of the literature showed that similar chemical states of carbon were found in the XPS C *1s* spectra of diamond-like carbon films deposited by microwave CVD [56] and thin diamond films synthesized by plasma-assisted CVD [57].

According to the fitting results, concentration of graphitic-like carbon in the surface layer is no more than 10 at% for all samples. The spectrum of Sample 1 exhibited a weak peak from *sp*^2^-carbon, and it was dominated by an oxygen-related peak at 286.0 eV (Figure 5a). Since oxygen was not introduced in the synthesis process, we propose that disordered graphitic-like surface carbon contained many dangling-bond defects that formed chemical bonds with oxygen atoms when the sample came in contact with laboratory air. It means that the surface of aggregates in Sample 1 are covered mainly by oxygenated carbon. This fraction is the smallest in Sample 3, whose surface is likely to consist of hydrogenated carbon (Figure 5c). Sample 2 is in an intermediate state, with remarkable oxygen-containing groups and *sp*^3^-hybridizied carbon (Figure 5b).

## 4. Discussion

HF-assisted CVD has been widely used for the synthesis of thin diamond films at low pressure because of its low cost, simplicity, reproducibility, mass production and uniform diamond deposition over a large area. However, the classical HF CVD method has a low nucleation density and a low growth rate of diamond for wide industrial applications. Here the diamond growth was suggested to be enhanced by using high-power thermal activation HF CVD and a high concentration of carbon precursors. The hot filament has two functions: to decompose the carbon precursor to carbon–hydrogen radicals and to generate large amounts of atomic hydrogen by the dissociation of molecular hydrogen. These two processes were separated in the space of the reactor using two gas jets. That helped to keep the activity of tungsten filament at high temperatures. The supply of hydrogen and carbon precursor as two separate gas jets at high speeds through the external and internal spaces of the tungsten spiral, heated above 2400 K, promoted the dissociation of hydrogen and methane, the fast delivery of free radicals to the substrate, and increased the diamond growth rate.

**Figure 6:**
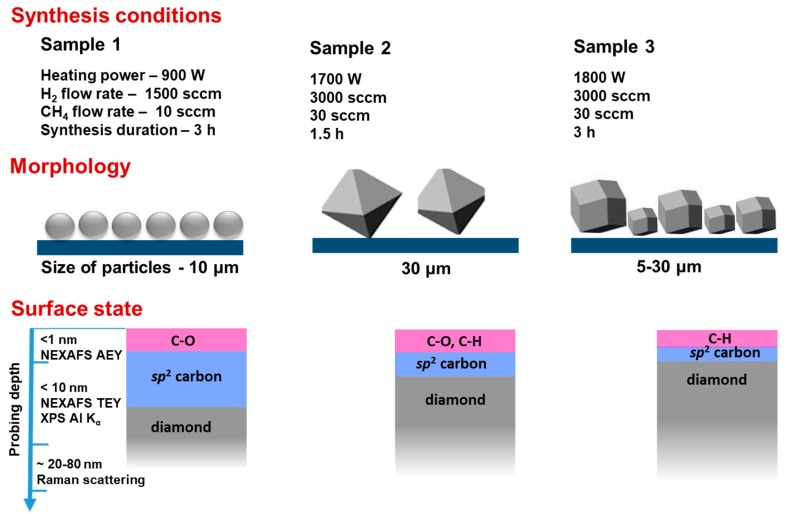
Schematic illustration of diamond film morphology and surface state for the samples grown at different heating power, methane and hydrogen flow rates, and synthesis duration.

We found the parameter window where the growth of diamond crystals is realized efficiently. Different characteristic tools were applied to study the morphology, composition, and structure of produced diamond samples. The findings on the morphology and surface state of the diamond crystals are schematically illustrated in Figure 6. In contrast to the classical HF CVD, where solid polycrystalline diamond films often cover the substrates uniformly [38], our films are not uniform and individual diamond crystals with sizes between about 6 and 30 μm are observed clearly. For many years seeding or abrading with diamond powder were used for better nucleation and deposition of diamond films. In our case, nanodiamond seeds used for the synthesis of Sample 1 did not improve the concentration, size, and quality of the diamond particles. Thus, we conclude that this trick is not suitable for the gas-jet deposition technique. Sample 1 was synthesis under a lower heating power of 900 W (estimated temperature was *ca*.2400 K) and a low concertation of hydrogen flow (1500 sccm) and methane flow (10 sccm). According to SEM and NEXAFS data, amorphous particles of size 10 μm with small-size diamond cores and thick amorphous carbon shells were grown in this sample. XPS revealed that defect sites occurred in the carbon surface of these particles, interacting easily with oxygen-containing molecules of air.

Samples 2 and 3 were synthesized at higher heating powers of 1700 and 1800 W (the estimated temperatures were ca. 2600 and 2700 K, respectively). Moreover, for both samples, the concentration of molecular hydrogen was two times higher than in the synthesis of Sample 1, while the concentration of methane was tripled. These changes caused an increase in the concentration of atomic hydrogen, which is believed to etch the *sp*^2^-carbon phase much faster than the diamond phase. Indeed, the diamond crystals with a more perfect crystal shape and structure were found in Sample 2 and 3. The phase identification of deposited films examined by Raman spectroscopy confirmed formation of diamond crystals of good quality.

It should be noted that our synthesis was carried out for a relatively short time (1.5 h for Sample 2 and 3 h for Sample 3), which is several times lower than that used in classical HF CVD methods. Despite the short synthesis time, 30 µm large diamonds grew. The growth rate for Samples 2 and 3 was estimated to be about 20 and 10 μm/h, respectively.These valuesare several times higher than growth rate in classical HF CVD. When the synthesis duration was increased to 3 h in Sample 3, the nucleation density increased, a denser diamond film was formed, and diamond crystals of smaller size were grown. We suggest that these changes in the morphology of diamond crystals are mainly determined by the mechanism of particle formation. A recent study of diamond growth mechanism in the HF CVD process revealed that diamond nuclei are formed in the gas phase and these nanoparticles formed in the gas phase are the building blocks for diamond films [58]. It can explain why, in the case of longer synthesis of Sample 3, the restoration of dimples and imperfections in diamond crystals, as well as the nucleation of new crystals onto the substrate surface and their growth happened. We believe that a solid diamond film could be grown by increasing the duration of the synthesis.

## 5. Conclusions

Diamond films were synthesized using the deposition of thermally activated high-speed jets of methane and hydrogen, which were spatially separated. Such an experimental approach allowed increasing the activator power without carbidization of its surface and the concentration of the atomic hydrogen. This contributed to the dissociation of hydrogen and methane, increasing the concentration of hydrogen and methyl radicals, which are required for the successful growth of diamonds. The diamond films grown at a different power of the heating activator, different flow rates of methane and hydrogen, and the different duration of the synthesis have been investigated by SEM, Raman scattering, XPS, and NEXAFS spectroscopies. It was shown that the diamond micro-crystals of 30 μm in size were grown on the Mo substrates when the power of heating was 1700–1800 W, which corresponded to the temperature of the tungsten heater of ca. 2700 K. While spherical microparticles with a large fraction of oxygenated graphitic-like carbon were grown at the low heating power of 900 W (2400 K) and twice lower hydrogen and methane flow rates, an increase in the synthesis duration from 1.5 to 3 h improved the quality of the crystals and increased their density on the substrate. Moreover, this caused the change of an octahedron shape of the diamond crystals to a rhombic-dodecahedron one. The diamond growth rate was estimated to reach 20 μm/h. The concentration of surface *sp^2^*-hybridized and oxygenated carbon decreased with an increase in the heating power, the flow rate of hydrogen and methane, and the duration of the synthesis. The diamond films with surface coatings containing *sp*^2^-hybridized and hydrogenated carbon of ~2nm thick were obtained. Thus, we demonstrated that by changing the parameters of the synthesis, the diamond films with different morphology, density, and chemical states of the surface can be synthesized. Controlled synthesis with specified structural and surface properties of diamond is essential for their further thermophysical, optic, and electronic applications.

## Figures and Tables

**Table 1:** Heating power (P), flow rate of hydrogen (R_H_) and methane (R_m_), substrate temperature (T_s_), presence of diamond seeds, and synthesis duration for Samples 1–3.

Sample	P, W	R_H_, Sccm	R_m_, Sccm	Substrate		Duration, h
				Diamond Seeds	T_s_, K	
Sample 1	900	1500	10	yes	1273	3
Sample 2	1700	3500	30	no	1273	1.5
Sample 3	1800	3500	30	no	1073	3
